# Ultrasound imaging of the lactating breast: methodology and application

**DOI:** 10.1186/1746-4358-4-4

**Published:** 2009-04-29

**Authors:** Donna T Geddes

**Affiliations:** 1 Biomedical, Biomolecular and Chemical Sciences, Faculty of Life and Physical Sciences, The University of Western Australia, Western Australia, Australia

## Abstract

Ultrasound imaging has been used extensively to detect abnormalities of the non-lactating breast. In contrast, the use of ultrasound for the investigation of pathology of the lactating breast is limited. Recent studies have re-examined the anatomy of the lactating breast highlighting features unique to this phase of breast development. These features should be taken into consideration along with knowledge of common lactation pathologies in order to make an accurate diagnosis when examining the lactating breast. Scanning techniques and ultrasound appearances of the normal lactating breast will be contrasted to those of the non-lactating breast. In addition ultrasound characteristics of common pathologies encountered during lactation will be described.

## Background

The lactating breast produces milk of a complex composition that is tailored for the optimal growth and development of the term infant [1], yet the knowledge regarding pathology and treatment of the lactating breast is limited compared to that of the non-lactating breast. Ultrasound imaging provides a non-invasive method of investigating the breast during lactation and this paper will review ultrasound techniques used during lactation along with normal and abnormal appearances of the lactating breast.

In the last 20 years imaging modalities have become more sophisticated however research has focused extensively on the abnormal non-lactating breast and little attention has been given to the normal and abnormal lactating breast. Mammography of the lactating breast is limited due to increased glandular tissue and the secretion of breast milk [2] causing an increase in radio-density that makes the radiographs difficult to interpret [3]. Galactography (the injection of radio-opaque contrast media into the duct orifice at the nipple and subsequent radiography) has illustrated only a portion of the ductal system, and few studies have examined lactating women. This procedure risks the introduction of pathogens into the breast and is therefore inappropriate for investigation of the lactating breast. To date both Computed Tomography (CT) and Magnetic Resonance Imaging (MRI) have had little to offer in elucidating pathology in the lactating breast. A recent report using MRI illustrated a duct after its injection with contrast [4] and another demonstrated dilated ducts and a high proportion of glandular tissue in seven lactating women [5]. However it is likely these modalities may provide much more useful information in the future. In the past, ultrasound investigation of the lactating breast has been limited for the same reasons as mammography; increased density of glandular tissue and the accumulation of milk [6]. More recently, however, malignancies have been confirmed during pregnancy and lactation with both mammography and ultrasound [7]. Ultrasound has undergone enormous technical advances that have improved the resolution of the images dramatically thus allowing imaging of very small structures within the breast. Ultrasound has the added advantage of being non-invasive thus allowing the breast to be examined without distortion. It follows that ultrasound would be the initial modality of choice for investigation of the lactating breast [8] however this requires a sound knowledge of breast anatomy and pathology and the development of imaging techniques unique to lactation. This paper describes the ultrasound technique used to investigate the anatomy of the lactating breast, current findings as well as breast pathologies associated with lactation.

### Gross anatomy of the lactating breast

**Figure 1:**
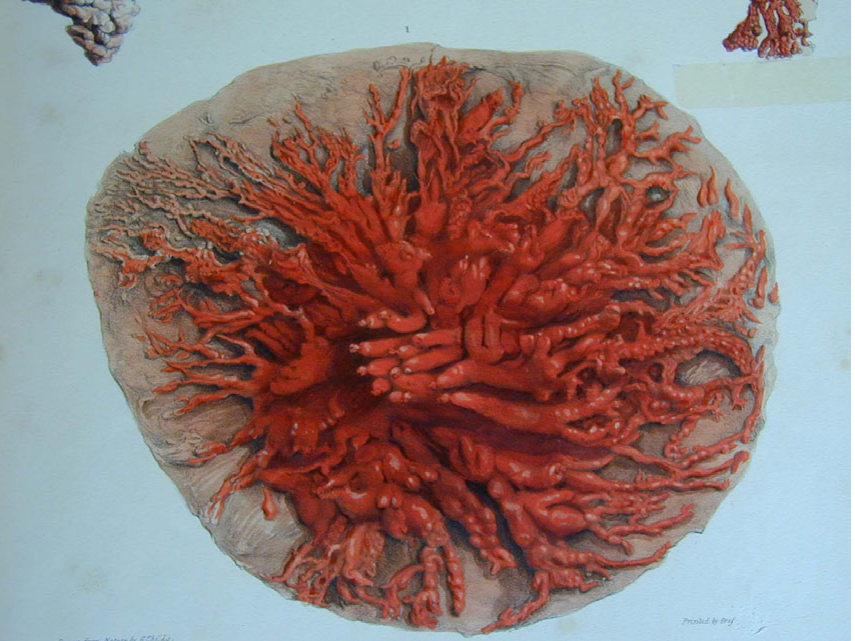
**Cooper's illustration of the ductal system of the lactating breast**. The breast of a woman who died during lactation, was injected with coloured wax and dissected.

**Figure 2:**
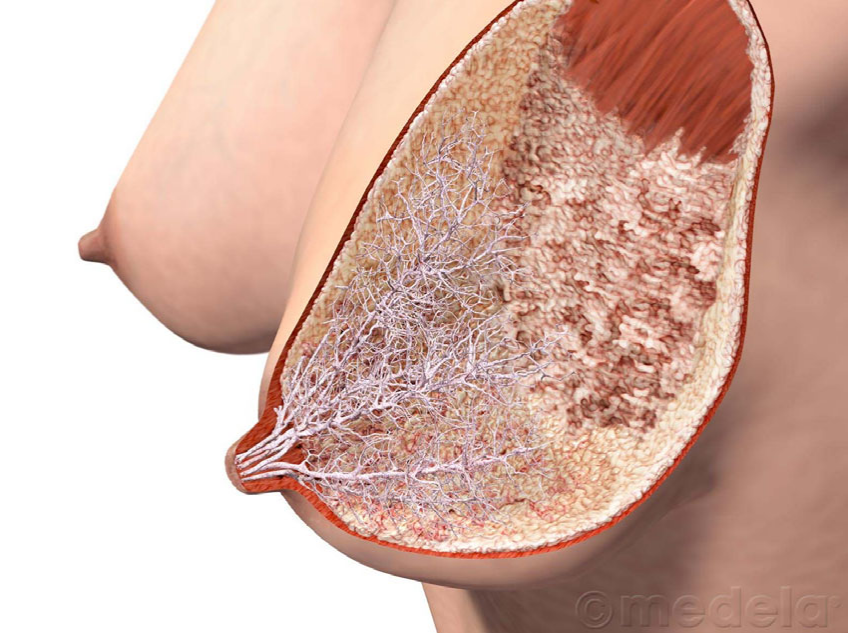
**Artist's illustration of the ductal system of the lactating breast based on ultrasound studies of lactating breasts**. (Reproduced with permission from Medela AG).

Standard descriptions of the human mammary gland are based on Cooper's dissections of the breasts of women who died during lactation (Figure 1) [9]. Recently Ramsay and colleagues re-investigated the anatomy of the lactating breast using high-resolution ultrasound [10]. We found fewer main ducts (mean 9; range 4–18) compared with the quoted 15–20 of conventional texts [11] which is in agreement with both Love and Barsky's [12] observations (mean 5; range 1–17) and Going and Moffatt's [13] dissection of one lactating breast (four patent ducts). Interestingly Cooper found seven to twelve patent ducts in a lactating cadaver although he could cannulate up to 22 ducts [9]. In addition we did not observe the typical sac like 'lactiferous sinus'. Instead ductal branches draining glandular tissue immediately below the nipple often merged into the main collecting duct very close to the nipple (Figure 2) [10]. An additional study showed that the milk ducts in the lactating breast only distend at the time of milk ejection, accommodating the transport of milk to the infant rather than storing milk for removal [14].

It is widely believed that the predominant tissue in the lactating breast is glandular. Ultrasound observations made throughout pregnancy show that the proportion of glandular tissue in the breast increases, although at six to twelve weeks adipose tissue was the most prevalent tissue in 20% of women [15]. Using a semi-quantitative ultrasound measurement of the glandular and adipose tissue in lactating Caucasian mothers it was found that there was approximately twice as much glandular tissue as adipose tissue in the lactating breast. However, the proportion of these tissues were highly variable with up to half of the breast comprised of adipose tissue in some women and conversely up to 80% of the breast composed of glandular tissue in others [10]. In addition it was found the amount of fat situated between the glandular tissues was highly variable which has also been observed in the non-lactating breast [16].

### Ultrasound equipment

#### Technical requirements

Breast ultrasound requires the highest resolution of almost all imaging procedures. In particular it requires high resolution of the near field (subcutaneous portion of the breast). The appropriate transducer is an electronically focused linear array with a frequency of 7–12 MHz with multiple focal zones to increase resolution of the area of interest [17]. However, in the case of the large lactating breast a 5 MHz probe may be desirable to both increase penetration of the breast and improve focusing at depth. Features that will improve imaging are: continuous electronic focusing, broad bandwidth and short pulse width. More recent developments such as coded harmonics and spatial compounding improve contrast resolution thus providing more detailed images of the structures of the breast.

#### Ultrasound settings

The time compensation curve (compensates for the normal attenuation of the sound waves in the tissue) ranges between a gentle slope for predominately fatty breasts to a steep slope for dense breasts. The gain setting compensates for attenuation without discriminating for depth thus amplifying all of the returning echoes [8]. Too high a setting will eliminate visualization of small structures and reduce the demarcation between adipose and glandular tissue. Too low a gain setting will result in the fat in the breast being displayed as anechoic (devoid of echoes or very dark/black) rather than hypoechoic (appears a darker shade of gray compared to surrounding tissues). One or two focal zones are used to improve resolution of the image, by narrowing the ultrasound beam, at selected depths of insonation. The power setting should be high enough to ensure adequate visualisation of all the tissues of the breast from the skin to the pectoral muscle [8,17]. Some ultrasound systems default to low power settings therefore one may need to increase the power before choosing a lower frequency transducer [18].

### Scanning technique

#### Patient position

When investigating the *non-lactating breast *for abnormalities the patient is often placed in the posterior oblique position with the breast to be examined raised. The objective of this position is to flatten the breast and bring the internal structures more parallel to the ultrasound beam. Thus the degree of obliquity depends on the size and shape of the breast and may vary during scanning. Upright positions are used occasionally to determine if there is either floating debris or dependent levels within cystic lesions. For the *lactating breast *it may be necessary to use a combination of oblique and upright positions to adequately examine the entire breast, particularly in women with very large breasts. Warm ultrasonic gel is used for scanning to enhance the transmission of sound through the skin into the breast and maintain good contact [3,18,19].

#### Compression

**Figure 3:**
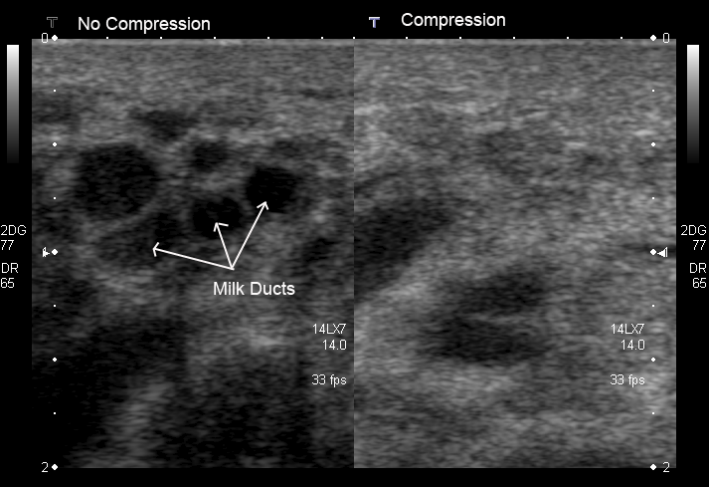
**Cross-sectional ultrasound image of milk ducts in the lactating breast**. On the left image, milk ducts appear as oval hypoechoic (black) structures. On the right image, milk ducts have collapsed under minimal to moderate compression with the ultrasound transducer.

Moderate compression of the *non lactating breast *during scanning is often used for improving both image quality, by changing the orientation of normal tissue so that it is perpendicular to the insonating beam, and visualization of small masses located deep within the breast [18,20] However, mild to moderate compression of the *lactating breast *will either compress or obliterate milk ducts thereby hindering visualization. It is prudent therefore to use moderate compression of the breast when targeting lesions but light compression when investigating the ductal system for abnormalities in the lactating breast (Figure 3).

#### Palpation

Ultrasound can be targeted to the area of a palpable abnormality in both the *non-lactating *and *lactating breast*. Location of the abnormality and simultaneous scanning should elucidate any distortion of the normal structures of the breast. When no abnormality is detected comparison to the opposite breast may be useful. Further investigation should be considered in the absence of ultrasound changes.

#### Scanning planes

The aim of the ultrasound examination of the breast is to survey the entire breast for abnormalities. When an abnormality is detected targeted ultrasound is employed.

**Figure 4:**
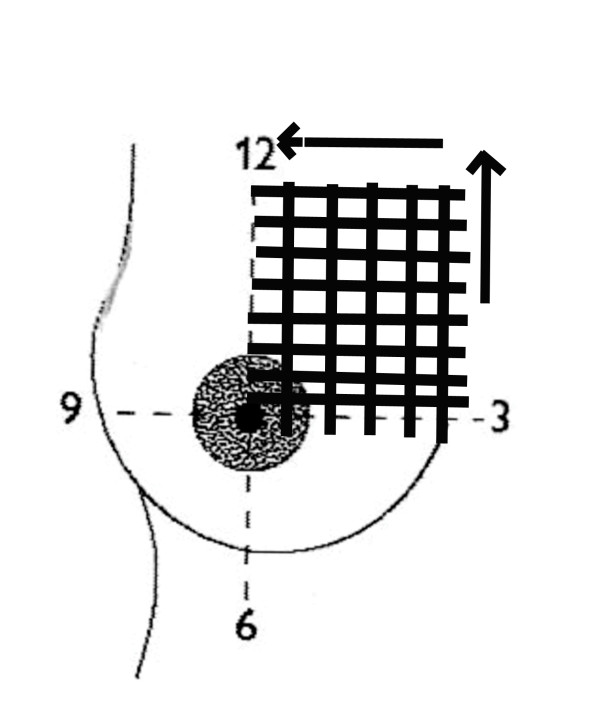
**Quadrant ultrasound scanning technique of the breast**. The breast is divided into four quadrants as shown and then each quadrant is scanned both vertically and horizontally. Care should be taken that all scans overlap to ensure scanning of the entire breast.

**Figure 5:**
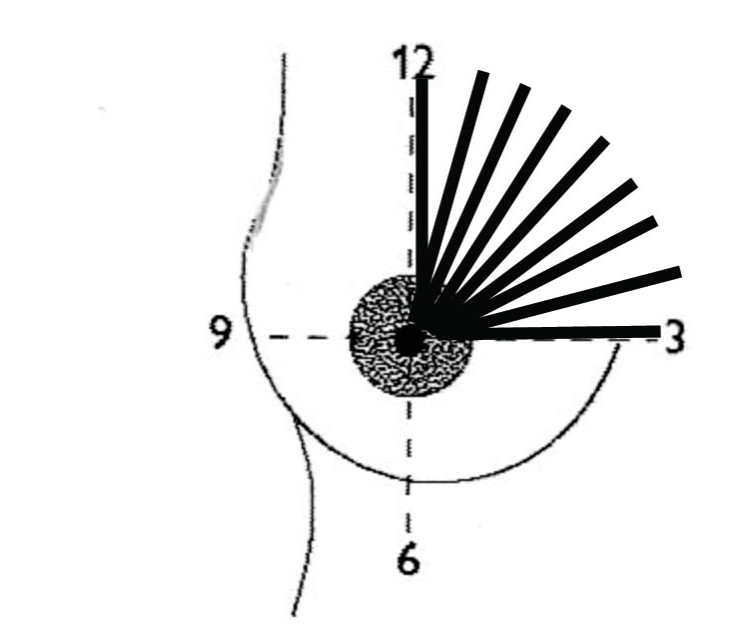
**Radial ultrasound scanning technique of the breast**. The breast is divided into four quadrants. Each quadrant is scanned in a radial fashion to accommodate the arrangement of ducts in the breast.

The real-time survey of the breast can be made using several different approaches. Commonly the breast is divided into quadrants and each quadrant is scanned using transverse and longitudinal planes ensuring that they overlap (Figure 4). Radial and anti-radial scanning planes are often favoured to demonstrate normal ductal anatomy particularly in the nipple-areola region (Figure 5) [3,18,21].

However a more radial and flexible approach is required in the lactating breast to interrogate the ductal system as the ducts have proliferated and often display an erratic course. Lobes are indistinguishable due to the intertwining nature of the ducts and lobules. If an abnormality is detected, targeted ultrasound using multiple planes and palpation, if possible should be performed. Labelling of images can be made with annotation (Clock Method) and/or body markers.

Assessment of the proportion of adipose and glandular tissue in the *non-lactating *breast is generally subjective with classifications one to four being made according to the proportion of echogenic tissue (parenchyma). Grade one represents mainly adipose tissue and with grade four the breast is predominantly comprised of echogenic tissue [22]. Ramsay and colleagues have developed a semi-quantitative method to assess/estimate the distribution of glandular and adipose in the *lactating *breast [10]. Using the clock face method images of the breast tissue are documented along eight radii (12.00, 1.30, 3.00, 4.30, 6.00, 7.30, 9.00 and 10.30 o'clock) of the breast. The images are taken sequentially along the particular axis from the base of the nipple to the outer portion of the breast until the glandular tissue is no longer visualized. Three to four images are documented along the radius. Each image includes all of the breast tissue from the skin to the chest wall and the full extent of glandular tissue from the nipple to the periphery of the breast. Measurements are made of the depth of glandular tissue (G), subcutaneous (S), intraglandular fat (I) and retromammary fat (R) at 30 mm intervals along the 8 radii of the breast from the base of the nipple. The thickness of each tissue is summed for the axis. The cumulative total of each tissue in the entire breast is therefore the sum of the total tissue measurements of each axis. For example total subcutaneous fat is given as:



S = Subcutaneous fat.

To describe the proportion of tissues within the breast, cumulative totals of all tissues are calculated (T).



Where G_TOT_, S_TOT_, I_TOT _and R_TOT _represent the sum of all depth measurements for all of the breast tissues made at 30 mm intervals for all 8 radii. Results are expressed as totals of the tissue in millimetres and as a percentage of the total tissue of the breast [10].

#### Nipple

**Figure 6:**
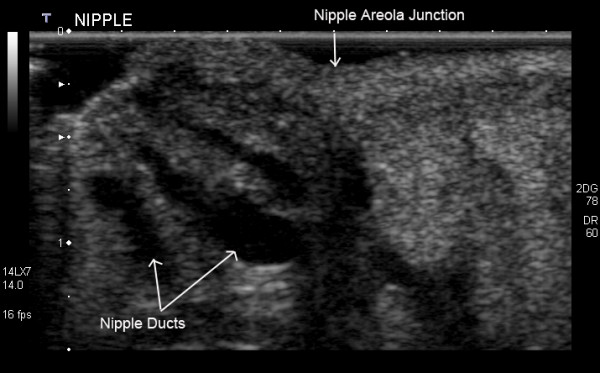
**Ultrasound image of the nipple of a lactating breast**. Ducts can be observed in the nipple as hypoechoic tubular structures. Visualization depends upon both the size of the duct and the resolution of the ultrasound equipment.

When obstruction of milk flow is suspected such as with nipple piercing or previous surgery special consideration should be given to scanning the nipple-areola area. Warm gel is advisable to avoid contraction of the muscle of the areola and nipple. Due the uneven and fibrous nature of the nipple distortion of the ultrasound beam may occur resulting in posterior acoustic shadowing rendering visualisation of the parenchyma behind the nipple poor [3,17]. Either the application of extra gel and pressure or angling around the nipple will ensure satisfactory documentation of this area [17,23]. We have found that an adapted version of rolled-nipple technique most useful in visualization of the ducts within the nipple (Figure 6) [18]. Stavros recommends placing the index finger on one side of the nipple and placing the probe on the other side of the nipple thus rolling the nipple onto the finger [18]. This re-orientates the nipple ducts so that they are perpendicular to the ultrasound beam thus improving resolution. Since mothers' nipples tend to enlarge during pregnancy and lactation, the nipple can often be re-orientated with the transducer and frequently the upright position can facilitate scanning.

### Normal ultrasonic appearances of the breast

The subcutaneous fat appears as a hypoechoic layer of tissue beneath the skin lines. Cooper's ligaments run between the superficial and deep fascia of the breast providing a framework for the parenchyma and appear as echogenic bands running obliquely from the posterior of the breast to the skin. The curved and fibrous nature of the ligaments may reflect the beam causing posterior shadowing. Changing the transducer pressure and angle will either reduce or eliminate this artefact [23,24]. The superficial fascia of the breast is occasionally seen as another thin echogenic line below the skin [24].

There is a wide range of ultrasonic patterns of the breast depending on the amount of fat interspersed throughout the glandular tissue. Generally the adipose tissue is hypoechoic with respect to the echogenic glandular tissue but is sometimes isoechoic. Ducts appear as small hypoechoic linear structures that are larger under the areola becoming progressively smaller towards the periphery of the breast [25]. Echogenicity of the duct can vary depending on both the surrounding tissue and the luminal contents [18]. The main ducts are arranged radially and two to three ducts can be identified merging with the main duct. Duct diameters above two to three millimetres are considered enlarged and indicative of ductal ectasia [26,27] or may be related to mastalgia [28], however a range of duct diameters from 0.6 to 4.4 mm have been measured in asymptomatic women [28]. Ducts of the *non-lactating *breast are generally not distorted by compression, unless containing fluid such as blood, and can be distinguished from vessels by the use of Colour Doppler Imaging. Colour Doppler Imaging is useful for suspicious lesions within a duct as they may exhibit vascularity [27]. Normal terminal ductolobular units can be imaged as isoechoic structures (same echogenicity as the fat) shaped like a tennis racquet hence are only visible when surrounded by the more echogenic fibrous tissue [18] therefore identification is variable. Some authors believe each of the lobes (segments) of the non-lactating breast can be imaged with ultrasound [25] despite the inability of surgeons to remove a lobe as a distinct entity [11]. Alternatively others refer to the glandular area as the mammary zone [18]. Difficulty discerning lobes is very likely due to their intertwined nature [2], however, the pattern of glandular tissue is observed more clearly by ultrasound than by mammography [22]. The retromammary fat appears as a hypoechoic layer above the pectoralis muscles that displays a typical fibrillar pattern.

**Figure 7:**
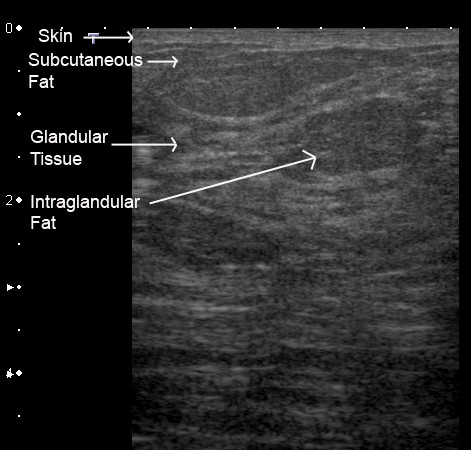
**Ultrasound image of the lactating breast**. The skin is displayed anteriorly as an echogenic line. Glandular tissue is hyperechoic and the fat more hypoechoic compared to the glandular tissue. Note that there is moderate amount of fat within the parenchyma of the breast.

**Figure 8:**
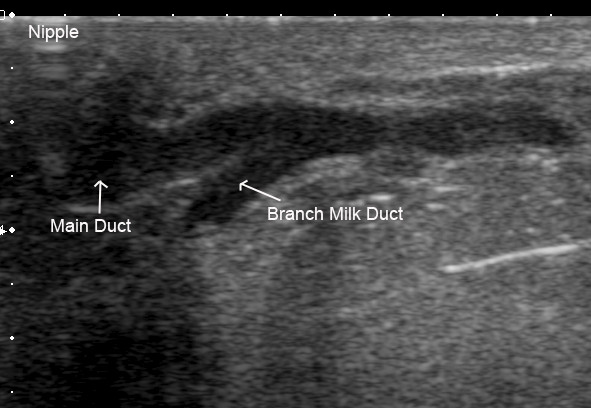
**Ultrasound image of a milk duct in a lactating breast**. The main duct is 8 mm long and 2.4 mm in diameter. The branch marked is 1.7 mm in diameter. This branch is draining glandular tissue directly under the nipple.

**Figure 9:**
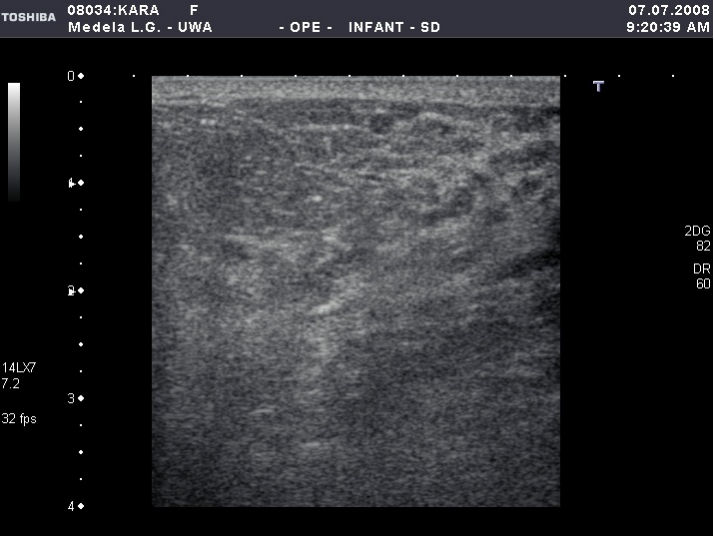
**Image of the left lactating breast at position 12 o'clock prior to a breastfeed**.

**Figure 10:**
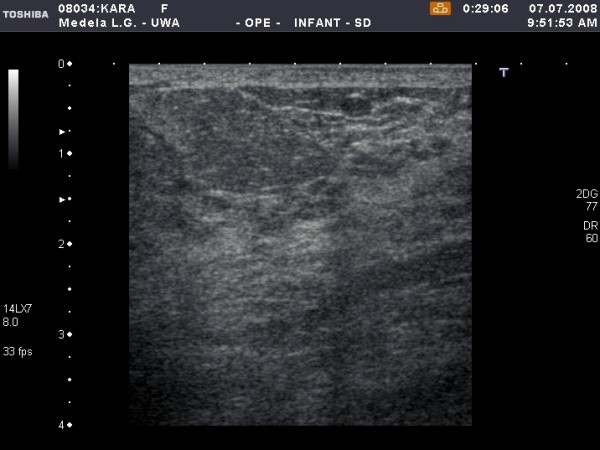
**Image of the left lactating breast at position 12 o'clock after a breastfeed**. The infant removed 58 g of milk. Ultrasonically there is a decrease in both the echogenicity and thickness of the glandular tissue compared to Figure 9.

Tissues of the *lactating breast *have similar echogenicity to that of the non-lactating breast with some exceptions (Figure 7; Table 1). The milk ducts of the lactating breast are on average relatively small (2 mm) with a wide range (0.9 to 10 mm) and branch close to the nipple thus not displaying large reservoirs of milk beneath the nipple (Figure 8). In addition the milk ducts compress easily under relatively little pressure (Figure 3) [10]. Furthermore, at milk ejection the milk ducts expand and milk flow can be observed within the duct. Duct dilation may be substantial (Additional file 1) or minimal (Additional file 2). Milk flow appears as echogenic flecks that result from reflection of the fat component of milk [14]. Milk ejection occurs during stimulation of the nipple in both suckling and pumping however it can also occur spontaneously. Conditioning of the milk ejection reflex is common and may be initiated by either the mother thinking of her infant or in response to her infant's cry [29]. Milk ejection may be accompanied by sensations in the breast and/or leaking of milk from the nipple. The echogenicity of the glandular tissue becomes more marked as more milk is synthesised and increasing amounts are stored in the breast (Figures 9, 10). Furthermore the breast becomes increasingly tense as it fills allowing limited compression thus impeding adequate penetration of the breast by the ultrasound beam. It may be prudent to ask the mother to either feed her infant or express milk prior to scanning in order to enhance imaging in these circumstances.

**Table 1:** The ultrasonic appearances of the structures of the non-lactating and lactating breast

Structures of the breast	Non-lactating breast	Lactating breast
Adipose tissue	Hypoechoic, variable Large breasts often contain a large proportion of adipose tissue	Hypoechoic, variable Large breasts often contain a large proportion of adipose tissue

Milk ducts	Hypoechoic/isoechoic Echogenic walls may be visible Generally non-compressible Do not distend 2 mm or less (>2 mm considered ductal ectasia)	Hypoechoic, can contain echogenic flecks representing milk fat globules Echogenic walls may be visible Easily compressible Distend at milk ejection Resting state – 2 mm (1–10 mm)

Skin	Hyperechoic (1–3 mm)	Hyperechoic Thicker in the areolar region

Coopers ligaments	Hyperechoic	Hyperechoic

Stromal fibrous tissue	Hyperechoic/isoechoic	Predominantly hyperechoic – tends to be more echogenic with more milk in the breast

Arteries and veins	Hypoechoic	Hypoechoic

### Blood flow to the lactating breast

The majority of the blood is supplied to the breast by two major arteries, the Internal Mammary Artery (IMA) and the Lateral Thoracic Artery (LTA). The IMA supplies the breast via the posterior and anterior medial branches and the Lateral Thoracic Artery supplies the lateral portion of the breast via the lateral mammary branch. Cooper depicted three anterior branches of the IMA, however he found most often that one branch located at the second intercostal space was larger and thus provided more blood to the gland compared to the others [9]. However iterations of Cooper's work has lead to a more extensive arterial network that includes branches of both the intercostal arteries and the thoracoacromial artery [30].

During pregnancy mammary blood flow increases to double pre-pregnancy levels by 24 weeks and then remains constant during lactation [30,31]. As with the non-lactating breast Geddes has shown that there is a wide variation between women in the proportion of blood supplied by each artery and there is little evidence of symmetry between breasts [32]. Along with an increase in blood flow, the superficial veins of the breast also become more prominent during pregnancy and lactation [32].

The 24 hour mammary blood flow required to produce one litre of milk in women is similar to that of other species (500:1). Currently no relationship between blood flow and milk production has been demonstrated in women. However, within a mother mammary blood flow is markedly reduced in a gland that is synthesising little milk compared to one producing a normal volume of milk. For example, in cases of unilateral hypoplasia and obstruction of milk flow due to nipple piercing mean blood flow velocities of the IMA and LTA have been shown to be reduced by half to two-thirds compared to the breast producing copious amounts of milk [32].

### Doppler ultrasound of the lactating breast

Many attempts have been made to determine if Colour Doppler Imaging can differentiate between benign and malignant masses with more accuracy than B-mode imaging alone. Results have been conflicting mainly due to many benign lesions exhibiting some vascularity [33].

### Ultrasound Doppler technique

**Figure 11:**
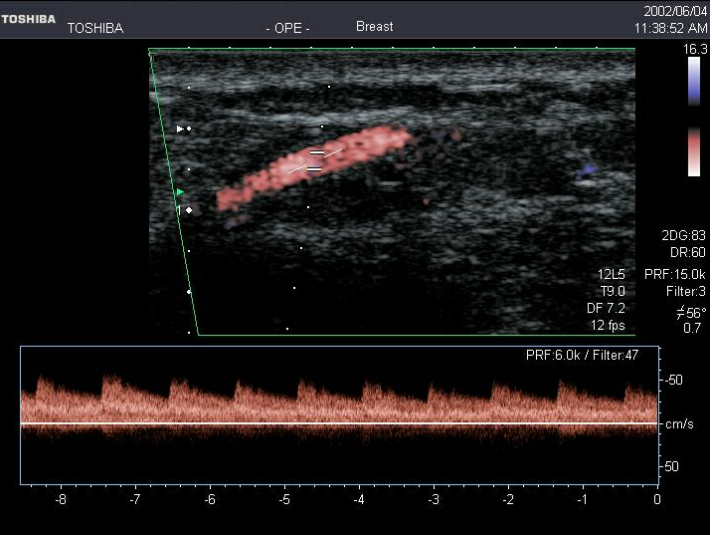
**The origin of the anterior mammary branch of the Internal Mammary Artery imaged with Colour Flow Doppler Imaging**.

The dominant mammary branch of the IMA can be located by positioning the transducer in a transverse plane alongside the sternum and making a sweep scan from the second to the sixth intercostal space. Colour Doppler imaging is essential to locate the IMA, which appears as a circular hypoechoic area between the rib spaces deep to the pectoral muscle. The probe is then rotated until the long axis of the branch of the IMA is imaged passing through the rib spaces towards the mammary gland (Figure 11) [34,35]. Doppler flow measurements are advised to be taken near the origin of the branch both distal to the pectoral muscle and removed from the parenchyma of the mammary gland. The mammary branch of the LTA can be located laterally and superiorly to the breast near the axilla. Generally settings for Colour Doppler are those typically used for low flow vessels, for example the velocity range can be as low as 4.5 cm/s. Steering of the Colour beam will facilitate detection and interrogation of vessels that are oriented almost parallel to the beam.

### Normal ultrasonic appearances and blood flow parameters

The arteries and veins of the breast can be visualized and assessed with Colour Doppler ultrasound. In addition veins are occasionally imaged as anechoic tubular structures that compress with gentle pressure. During breastfeeding blood flow decreases by 40–50% just prior to milk ejection and then increases in the following one to two minutes [36]. Spontaneous milk ejections can occur during scanning which may affect Doppler measurements. Common signs of milk ejection are leaking of milk from the nipple, sensations in the breast of pins and needles, pain pressure and sometimes maternal feelings of warmth and nausea [29,37].

Little research has been carried out with regard to normal mammary blood flow parameters in both the lactating and non-lactating breast. Current knowledge of known parameters is given in Table 2. If the ultrasound machine used does not automatically calculate flow rate (volume of blood moving through the artery per unit of time) this can be manually calculated with the equation given below:

**Table 2:** Doppler blood flow parameters for the mammary branches of the Internal Mammary Artery Branch (IMA) and Lateral Thoracic Artery (LTA) for both the non-lactating and lactating breast

Mean blood flow parameters	Non-lactating breast	Lactating breast
IMA Diameter (mm)	0.2 [68]	1.8

Systolic velocity (cm/s)	-	56

Diastolic velocity (cm/s)	-	25

Mean velocity (cm/s)	19 [68]	39

Flow volume (mL/min)	45.5 [68]	85

LTA Diameter (mm)		1.3

Systolic velocity (cm/s)	-	37

Diastolic velocity (cm/s)	-	16

Mean velocity (cm/s)	-	24

Flow volume (mL/min)	-	45

Pulsatility Index	2.2 (2) [69]	0.88

24 hour mammary blood flow (L)	-	170–200

- no data available



TMAX = Time average maximum velocity

Area = πR^2^

### Lymphatics of the breast

The lymph in the breast is drained by two main pathways; to the axillary [38] and internal mammary nodes [38,39]. The axillary nodes have been reported to receive more than 75% of the lymph from both the medial and lateral portions of the breast [40], whereas, the internal mammary nodes receive lymph from the deep portion of the breast [41]. Nevertheless there is a wide variation in the drainage of lymph from the breast and less common pathways have been demonstrated. Lymph may occasionally pass through either the interpectoral nodes [11] or lymph nodes in the breast parenchyma [42]. Sometimes direct drainage of lymph occurs to the supraclavicular nodes [42] and infrequently lymph may pass retrosternally into the contralateral internal mammary nodes. In addition lymph has been shown to drain into the posterior and anterior intercostal nodes [42].

### Normal appearances of the lymphatics of the breast

There has been little investigation of the lymphatic drainage of the lactating breast despite its importance in clinical conditions such as engorgement and mastitis.

Mammary nerves and normal lymphatics are not visualised on ultrasound, however when the lymphatics are dilated due to either inflammation or malignant invasion they become visible as very thin anechoic/hypoechoic lines running parallel and perpendicular to the skin in the subcutaneous tissues [3]. Lymph nodes are demonstrated in the breast and axilla as well defined oval masses with an echogenic hilum and hypoechoic cortex [43].

### Pathology of the lactating breast

Ultrasonic features of pathology of the lactating breast are summarized in Table 3.

**Table 3:** Ultrasonic characteristics of common pathologies of the lactating breast

Pathology	Ultrasonic appearances
Cyst	Margins – well circumscribed with thin smooth walls Centrally anechoic Posterior enhancement Edge shadowing No internal vascularity

Fibroadenoma	Margins – well defined or occasionally ill-defined Echogenicity – homogenous to heterogenous No posterior enhancement unless internal calcification is present Internal vascularity

Abscess	Margins – wide, indistinct, hypoechoic Echogenicity – predominantly echo-free to heterogenous Posterior enhancement No internal vascularity

Malignancy	Margins – irregular and ill-defined Echogenicity – heterogenous echogenicity Stellate appearance +/- posterior shadowing Internal vascularity

Galactocele	Acute – anechoic and simple or mainly anechoic with some diffuse echoes and multiloculated. Sub-acute – contain echoes of mild to moderate intensity Chronic – diffuse echogenicity ranging from moderate to highly echogenic Can be simple, multilocular and heterogenous Possible fat-fluid level Movement of the contents can be demonstrated by compression with the transducer Galactoceles are centrally devoid of blood vessels however flow may be demonstrated in the walls – use of colour Doppler can confirm this

Blocked duct	Focal – similar appearances to an acute galactocele, non-compressible. Diffuse – often an area of increased echogenicity associated with a palpable solid region. Occasionally a hypoechoic rim surrounds a more echogenic central region

Lactating adenoma	Margins – well circumscribed to ill-defined Echogenicity – hypo-, hyper or isoechoic Homo- or heterogenous Posterior enhancement or acoustic shadowing +/- internal vascularity

Engorgement	Increased echogenicity of the glandular tissue due to the large volume of milk in the breast. Severe engorgement may exhibit ultrasonic signs similar to mastitis (see below)

Mastitis	Early/acute phase: there may be no discernable ultrasonic changes in echogenicity breast tissues Skin – thickens and becomes more hyperechoic Cooper's ligaments and stromal fibrous tissue decrease in echogenicity Areas of inflammation frequently have increased blood flow Advanced stages: Skin thickening is prominent Distinction between different breast tissues disappears Breast thickness increases

#### Focal masses

**Figure 12:**
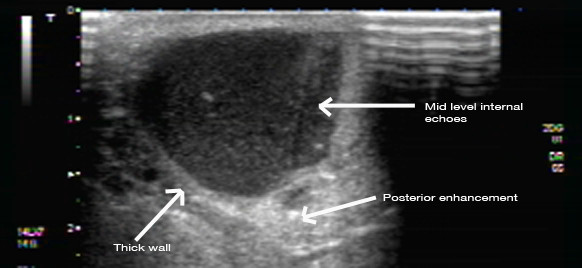
**Ultrasound image of a palpable lump behind the nipple of a pregnant woman**. The mass is thick walled with mid-level internal echoes and posterior enhancement.

**Figure 13:**
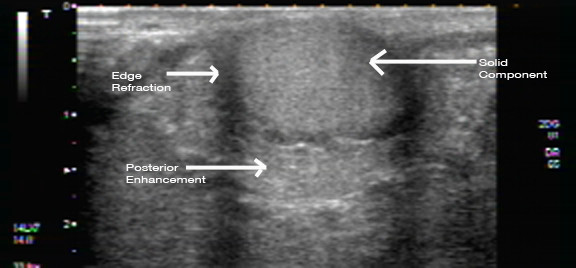
**Ultrasound image of a palpable lump behind the nipple of the pregnant woman in Figure 12**. The mass also contained an internal hyperechoic solid component with edge refraction and through transmission of sound.

While the texture of the breast changes during pregnancy and lactation persistent focal lumps are not considered normal and should be investigated appropriately. Furthermore some women may have pre-existing benign lesions prior to lactation and any noticeable changes in these areas warrant examination. In these instances ultrasound is usually the first investigation of choice. Mammography is less desirable owing to both the compression of the breast and the difficulty in diagnosis due to the increased density of radiographs caused by the proliferation of glandular tissue and the presence of milk. All lesions in the lactating breast display the typical ultrasonic features that would be expected in the non-lactating breast. However the size and location of the mass may cause obstruction of milk flow by compressing milk ducts particularly in the event of adjacent alveoli becoming very full of milk. In one case that presented at our laboratory multiple fibroadenomas were confirmed. Several of the lesions were located in the areola region. This mother and infant experienced breastfeeding difficulties which led to partial breastfeeding despite advice from a lactation consultant. Another woman who presented during pregnancy was experiencing leakage of colostrum from the left breast and no leakage from the right. A large complicated fluid filled mass behind the right nipple was detected and milk ducts were unable to be traced around the mass (Figures 12, 13). With this information an appropriate management plan for both the mass and lactation could be formulated prior to the birth.

#### Cysts

**Figure 14:**
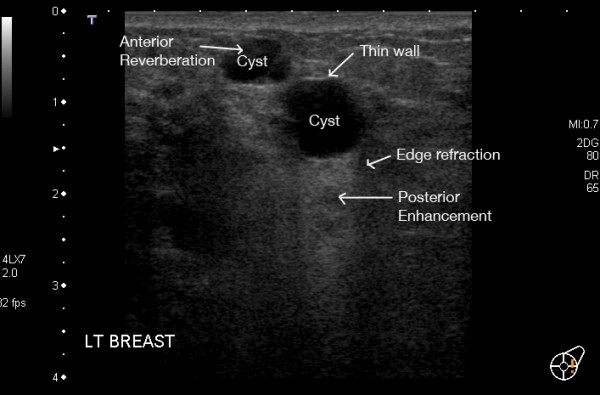
**Ultrasound image of multiple cysts in the left breast of a lactating woman**. The cysts display typical criteria such as thin walls, posterior enhancement, edge refraction and anterior reverberation.

Although not common, cysts are occasionally present in the lactating breast. They have the same ultrasonic characteristics as cysts in the non-lactating breast such as a well defined margin, internally echo-free, posterior enhancement and edge refraction (Figure 14). There should be no internal vessels present with Colour Doppler Imaging. Due to the increased echogenicity of the parenchyma of the lactating breast it may be more difficult to attain an echo-free centre [3,18].

#### Fibroadenoma

**Figure 15:**
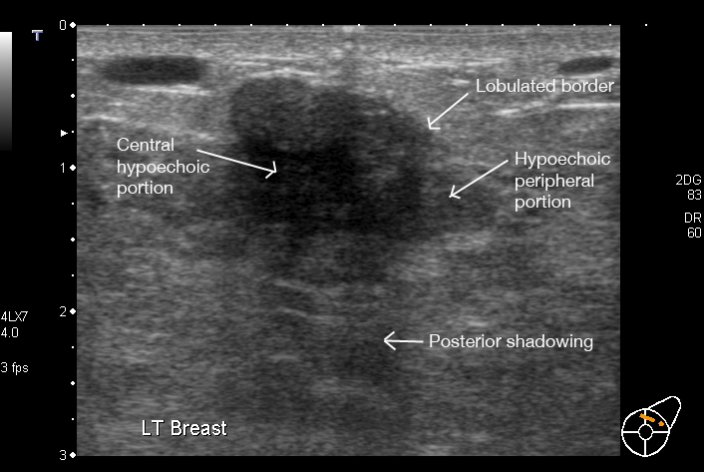
Fibroadenoma in a lactating breast – incorrect**. This fibroadenoma was diagnosed and investigated prior to pregnancy. It appears ultrasonically as a heterogeneous lobulated mass with reduced sound transmission.

**Figure 16:**
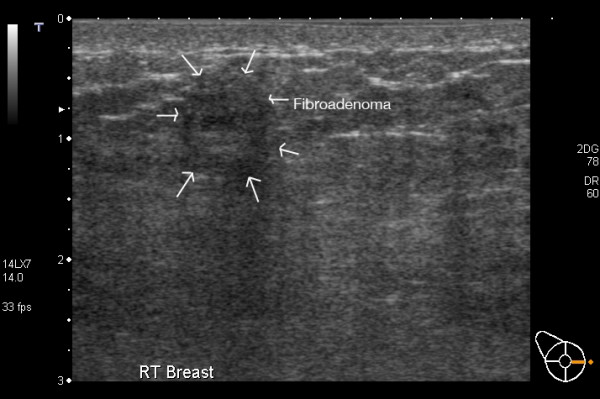
**Fibroadenoma in a lactating breast**. This fibroadenoma is difficult to image with ultrasound as it is almost the same echogenicity as the surrounding tissues.

Fibroadenomas persist and may enlarge during pregnancy and lactation in response to increased oestrogen [44]. There is a broad spectrum of ultrasonic appearances. Most often fibroadenomas are well-defined masses of either homogenous or heterogeneous echogenicity depending on their composition (Figure 15, 16). Most often they transmit sound thus not inducing posterior shadowing artefact. Depending on the age of the fibroadenoma calcification can be present and may or may not cause posterior shadowing [3,18]. Central blood flow may or may not be evident on Colour Doppler Imaging.

#### Galactocele

Galactoceles are dilated terminal ducts (ductules) comprised of a layer of epithelium and a layer of myoepithelium and are filled with milk. Their cause is thought to be the result of an obstruction of a milk duct by either a lesion or inflammation [45]. The echogenicity of the galactocele is dependent upon its stage as the protein in the milk denatures and fat emulsifies over time. Galactoceles tend to have well-defined, thin echogenic walls but may also present with an anechoic fluid rim. The internal echogenicity however varies from homogeneous mid-level echoes to heterogeneous echogenicity with or without accompanying fluid levels. Distal enhancement is present due to lack of acoustic attenuation provided by the milk. Echogenic areas with acoustic shadowing are believed to be inspissated contents [46]. Their shape may also depend upon the location in the breast. Aspiration under ultrasound guidance is diagnostic and therapeutic in cases of large galactoceles [47].

#### Blocked/plugged ducts

**Figure 17:**
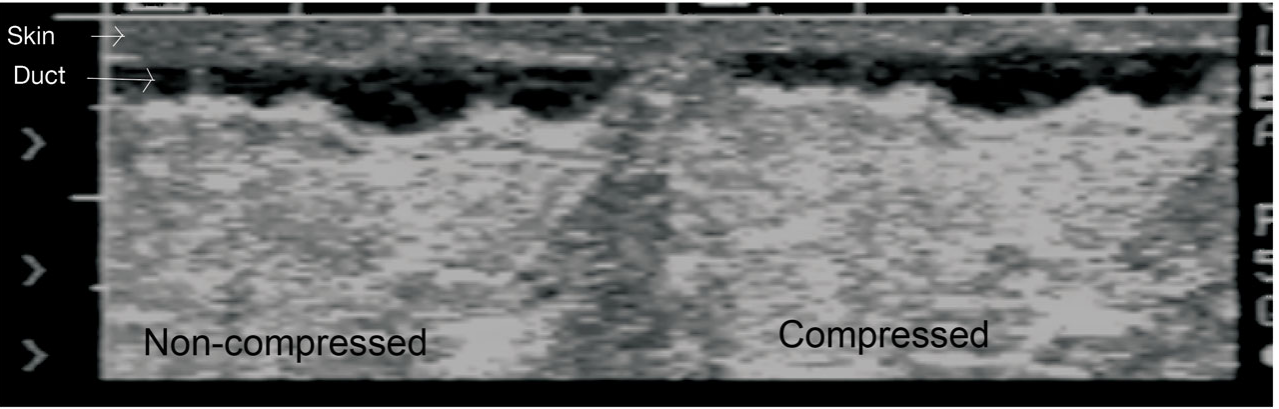
**This blocked duct presented as a discrete mobile palpable lump**. Ultrasonically it appears as a small hypoechoic tubular structure duct that is incompressible.

**Figure 18:**
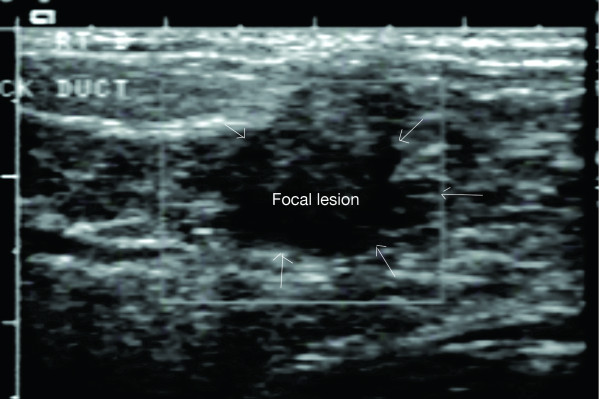
**This palpable blocked duct appears as a small focal heterogeneous area with irregular margins on ultrasound**.

**Figure 19:**
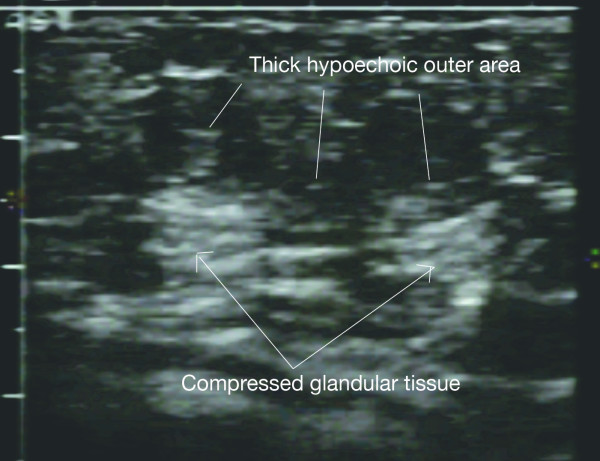
**Blocked duct presents as a large area of breast tissue of altered echogenicity on ultrasound**. The central area is increased in echogenicity and surrounded by a more hypoechoic rim. A large wedge shaped hardened area was felt on palpation.

Blocked ducts commonly present as a tender lump ranging from the size of a pea to a large wedge shaped area. They are not associated with either redness of the skin or maternal fever. Suspected causes include changes in infant feeding pattern, mechanical obstruction (underwire bra, restrictive clothing) and either scarring from previous breast surgery or infection [48]. More recently selective Secretory Immunoglobulin A deficiency has been identified in a mother with recurrent blocked ducts and more research is required to determine if this condition is causative [49]. Resolution generally occurs with conservative management involving massage of the nodular area and increased frequency of removal of milk from the affected breast by either breastfeeding or expression [50]. Ultrasound appearances range from a discrete incompressible mass (Figures 17, 18) to a diffuse echogenic area with a hypoechoic rim (Figure 19) associated with a hardened area of the breast [18]. Occasionally the blocked duct may appear as an incompressible duct that can be traced to the origin of the blockage, which may be at the nipple. Focal lesions should be monitored and fine needle aspiration considered should they not resolve with treatment. In cases of recurrent blocked ducts it would be pertinent to exclude an obstructing lesion [51].

#### Abscess

Abscesses reportedly occur as a complication of approximately three percent of mastitis cases in developed countries [52] and vary in their ultrasonic presentation. The margins of the abscess are often wide, indistinct and hypoechoic compared to surrounding tissues. The centre is fluid filled and the echogenicity ranges from hypoechoic to mixed echogenicity. Occasionally layers are visible within the abscess. Posterior enhancement is evident due to the fluid filled nature of the abscess and it will have limited compressibility [6]. Colour Doppler ultrasound imaging may assist with demonstrating internal blood flow in inflamed hypoechoic tissue thus ruling out an abscess [53]. Abscesses may be drained under ultrasound guidance however, follow up to ensure complete resolution is recommended in these cases [53-55]. More recently vacuum assisted drainage has shown to be successful in lactating women with recurrent abscesses [56]. Alternatively abscesses can be incised and drained surgically. Cessation of breastfeeding is not necessary during any of the treatments [57].

#### Lactating adenoma

Lactating adenomas are a relatively uncommon breast tumour that is often first recognized during either pregnancy or lactation. They develop from the inner most layer of alveoli which is comprised of lactocytes (secretory epithelium) [58]. Since there are a wide variety of ultrasonic appearances that include benign and malignant features a large core needle biopsy (LCNB) is often performed to obtain a diagnosis. LCNB is preferred to fine needle aspiration to reduce the possibility of false-positive diagnoses of malignancy. Many adenomas resolve after weaning however some women opt to have them surgically removed [44,59].

#### Breast cancer

The incidence of breast cancer in pregnant and lactating women varies from 1 in 3000 to 1 in 10000 women [60,61]. Symptoms often begin before or during pregnancy [61]. Unfortunately these cancers are often at an advanced stage as diagnosis is frequently delayed. In addition the increased mammary blood flow during pregnancy and lactation may accelerate the growth of the tumour [62]. The sensitivity of mammography is reduced due to the increased amount of glandular tissue and water content of the breast resulting in increased parenchymal density of the radiographs. However, ultrasound has been shown to be accurate in pregnant and lactating women with a focal mass [61].

Breast cancers in pregnant and lactating women exhibit the same typical features as would be expected in the non-lactating woman – a focal mass of heterogeneous or low echogenicity with irregular margins. Additional features such as posterior shadowing may or may not be present. In addition the axillary lymph nodes should be scanned to exclude metastases.

### Diffuse pathologies

#### Engorgement

A rapid increase in milk production occurs at secretory activation around day two to five postpartum [63]. Breasts can become quite tense and full at this stage. Symptoms resolve with frequent feeding and/or effective emptying of milk from the breast. Cold compresses may also assist in relief of the symptoms. Severe engorgement may lead to compromised milk supply, nipple trauma and mastitis [47]. Ultrasound appearances include an increase in echogenicity of the glandular tissue due to the large volume of milk in the breast. In addition the breasts are often tense and painful. Severe engorgement may exhibit ultrasonic signs similar to mastitis such as skin thickening and increased vascularity.

#### Mastitis

Mastitis is an inflammation of the breast and has been classified into two types: infectious and non-infectious. Non-infective mastitis can occur as a result of blocked ducts, engorgement or physical injury to the breast resulting in a localized inflammatory response [64]. Infective mastitis is a result of invasion of the breast by a pathogen most commonly *Staphylococcus aureus *however other species such as β-*haemolytic streptococci, Streptococcus faecalis *and *Escherichia coli *have been identified as causative organisms. The most common passage of entry is considered to be via nipple fissure due to trauma [65]. Indeed this is feasible considering retrograde milk flow is noted within the milk ducts during the latter half of milk ejection in the breast that is not fed or pumped from [14,66,67].

**Figure 20:**
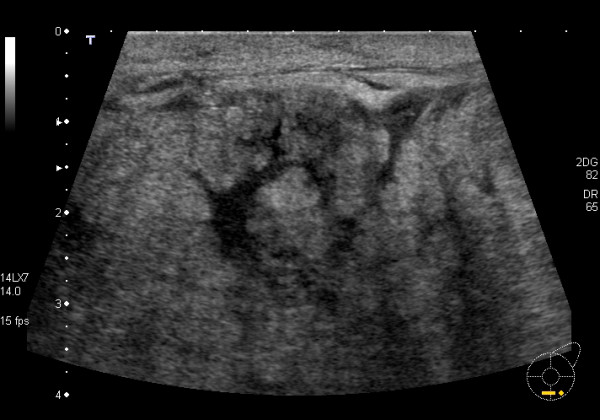
**Mastitis of a lactating breast**. The ultrasonic distinction of normal breast tissues is not evident. The parenchyma is markedly echogenic, ducts are non compressible and the skin lymphatics are visible. In addition the skin is thickened.

Ultrasonic appearances may vary due to the duration and extent of the inflammation. Very early in the acute phase there may be no discernable ultrasonic changes in echogenicity of the breast. Skin thickening can occur and the skin becomes more hyperechoic (brighter than the surrounding tissues). Normally hyperechoic structures such as the Cooper's ligaments and stromal fibrous tissue decrease in echogenicity and become more difficult to distinguish from adipose tissue. Areas of inflammation frequently have increased blood flow in the local vessels compared to the corresponding area in the contra-lateral breast. In advanced stages distinction between different tissues disappears, breast thickness increases and skin thickening is prominent (Figure 20). At this stage a lower frequency (5 MHz) probe may be necessary to penetrate the breast [18]. Serial monitoring will demonstrate a decrease in blood flow with resolving inflammation [6]. Although uncommon, ultrasonic appearances of inflammatory carcinoma may mimic mastitis and follow-up to ensure resolution should avoid misdiagnosis.

## Conclusion

Ultrasound imaging is the most appropriate initial investigation of the pathological lactating breast. However the mammary anatomy, increased density of glandular tissue, compressibility of milk ducts, raised mammary blood flow and the changes in mammary physiology associated with lactation should be taken into consideration when refining breast ultrasound scanning techniques. Furthermore knowledge of lactation-associated pathology will ensure more accurate diagnoses and treatment for lactating women.

## Competing interests

The author receives a salary as part of a research grant provided by Medela AG.

## Supplementary Material

Additional File 1 **Ultrasound video of milk ejection in the unsuckled breast during a breastfeed**. Substantial duct dilation accompanied by milk flow is evident at milk ejection. Milk is flowing towards the right upper corner of the image where the nipple is situated. Click here for file

Additional File 2 **Ultrasound video of milk ejection in the non-expressed breast during a pumping session**. Minimal duct dilation accompanied by obvious milk flow is observed at milk ejection. The nipple is situated in the upper right corner of the image. Click here for file
